# Clinical presentation and diffusion weighted MRI of acute cerebral infarction. The Bergen Stroke Study

**DOI:** 10.1186/1471-2377-9-44

**Published:** 2009-08-18

**Authors:** Halvor Naess, Jan C Brogger, Titto Idicula, Ulrike Waje-Andreassen, Gunnar Moen, Lars Thomassen

**Affiliations:** 1 Department of Neurology, Haukeland University Hospital, 5021 Bergen, Norway; 2 Department of Radiology, Haukeland University Hospital, 5021 Bergen, Norway

## Abstract

**Background:**

No large study has compared the yield of diffusion-weighted imaging (DWI) with clinical examination in order to differentiate lacunar stroke from other stroke subtypes. This differentiation is important for guiding further investigations and treatment.

**Methods:**

Consecutive patients admitted with cerebral infarction were classified according to the Oxfordshire Community Stroke Project scale. Based on DWI and CT stroke was classified as lacunar (LI) and non-lacunar (NLI). Acute ischemic lesion <1.5 cm and located in subcortex or in brainstem were classified as LI. All other infarctions were classified as NLI.

**Results:**

DWI was performed in 419 (69%) patients. Among patients with lacunar syndrome (LACS) 45 (40.5%) had NLI on DWI. All patients with total anterior syndrome (TACS) and 144 (88.3%) with partial anterior syndrome (PACS) had NLI on DWI.

**Conclusion:**

DWI is important among patients presenting with clinical symptoms suggestive of lacunar syndrome to differentiate between LI and NLI. On the other hand, there is good correspondence between TACS or PACS and NLI on DWI.

## Background

The three most common causes of cerebral infarction are large-vessel atherosclerosis, small vessel disease and cardiac embolism [1,2]. Histopathologic studies disclosed that small vessel disease causes subcortical infarctions <1.5 cm in diameter (lacunar stroke) [3]. None-lacunar infarctions comprising subcortical infarctions and brainstem infarctions ≥1.5 cm with or without involvement of the cortex and pure cortical infarctions tend to be associated with large-vessel atherosclerosis and cardiac embolism [4]. Secondary preventive treatment after cerebral infarction is dependent on aetiology and risk factors [5]. It is important to differentiate between lacunar and none-lacunar infarctions because the latter is often caused by cardiac embolism in need of long-term anticoagulation. It is unlikely that lacunar infarctions are caused by cardiac embolism [6,7]. Patients presenting with none-lacunar infarction may need extensive cardiac evaluation such as transesophageal echocardiography or repeated Holter monitoring. A previous study recommended that clinical presentation based on the Oxfordshire Community Stroke Project (OCSP) scale could be used to guide further investigation unless visible infarctions were present on brain imaging [8]. However, the studies comparing OCSP and brain imaging relied heavily on CT and MRI without diffusion weighted imaging (DWI) [8-11]. It is well-known that DWI is much more sensitive than CT as to detection of acute ischemic lesion [12].

In our experience, misclassification of the underlying aetiology is frequent when based on clinical findings and CT examinations. The aim of this study was to compare DWI with clinical presentation based on the OCSP scale as to differentiating between lacunar and non-lacunar infarctions in a large cohort of patients with acute cerebral infarction reflecting real-life clinical experience from a single centre.

## Method

All consecutive patients with acute cerebral infarction admitted to the Stroke Unit, Department of Neurology, Haukeland University Hospital in Bergen, Norway between February 2006 and March 2008 were prospectively registered in a database (The Bergen Stroke Study). Cerebral infarction was defined in accordance with the Baltimore-Washington Cooperative Young Stroke Study Criteria comprising neurological deficits lasting more than 24 hours because of ischemic lesions or transient ischemic attacks where CT or MRI showed infarctions related to the clinical findings [13].

CT was performed as soon as possible after admission to the hospital. It was the routine of our department to refer all patients with cerebral infarction to MRI unless there were contrary reasons such as pacemaker, none-consenting or unstable patient. DWI was performed as part of a routine MRI protocol for stroke patients on 1.5 Tesla Siemens Magnetom (Symphony). The DWI-sequence used was ep2d_diff_3scan_trace, with the following specifications of parameters: Field of view (FOV) 230 mm, Slice thickness 5 mm, TR 3200 ms, TE 94 ms. All CT and MRI scans were reviewed by a neurologist (HN) with long CT and MRI experience.

Isolated acute ischemic lesions on CT or MRI were defined as lacunar infarctions (LI) if <1.5 cm and located subcortical or in the brainstem [14]. All other acute ischemic lesions were defined as none-lacunar infarction (NLI). NLI comprised subcortical and brainstem infarction ≥1.5 cm, cortical infarction, mixed cortical and subcortical infarction and cerebellar infarction.

The National Institute of Health Stroke Scale (NIHSS) was used to assess stroke severity. NIHSS measurement was performed several times during the first 24-48 hours after admittance either by neurologist or experienced stroke nurse.

Clinical classification was based on the OCSP scale which includes lacunar syndrome (LACS), partial anterior circulation syndrome (PACS), total anterior circulation syndrome (TACS) and posterior circulation syndrome (POCS)[15] OCSP has good inter-observer reliability [16]. OCSP classification was performed by trained stroke neurologists (HN and UWA) within one week after stroke onset. Aetiology was determined by the Trial of Org 10172 in Acute Stroke Treatment classification (TOAST).

The study was approved by the local ethics committee. Informed consent was obtained from all patients.

### Statistics

Student's t-test, Fisher's exact test, Pearson chi-square test and logistic regression were used when appropriate. Stata 9.0 was used for analyses.

## Results

In total, 608 patients had cerebral infarction. All patients had either CT, MRI or both. Median time from stroke onset to CT was 4 h 52 min (interquartile range 12 h 23 min), and median time from stroke onset to MRI was 2 d 1 h 12 min (interquartile range 2 d 3 h 29 min). DWI was performed among 419 (69%) patients. Among patients <80 years 79.3% had DWI. Table 1 shows a comparison between patients with and without DWI. Patients who underwent DWI were younger, had lower NIHSS score on admission, and less often cardioembolic stroke. Logistic regression showed that undergoing DWI was independently associated with lower age (P < .001) and lower NIHSS score on admittance (P < .001), but not sex (P = .71).

**Table 1:** Characteristics of patients with acute cerebral infarction who did and did not undergo DWI examination (n = 608)

	DWI		Not DWI		P
		
	n	%	n	%	
Male	256	61	94	50	.010
Age (mean)	67.3 years		76.7 years		< .001
NIHSS score on admission (mean)	4.6		9.7		< .001
Atherosclerosis	65	15.5	19	10.1	.076
Cardiac embolism	90	21.5	69	36.5	< .001
Small vessel disease	78	18.6	21	11.1	.024
LACS	118	28.2	39	20.6	*
PACS	167	40.0	68	36.0	*
TACS	48	11.5	63	33.3	*
POCS	85	20.3	19	10.1	*

* P < .001 (Pearson chi-square test)

DWI: diffusion weighted imaging

NIHSS: National Institute of Health Stroke Scale

LACS: lacunar syndrome

PACS: partial anterior circulation syndrome

TACS: total anterior circulation syndrome

POCS: posterior circulation syndrome

DWI lesions were detected in 398 (95%) patients of the patients who underwent MRI. LI was detected in 108 (27%) patients and NLI in 290 (73%) patients based on DWI. Table 2 shows a comparison between patients with LI and patients with NLI based on DWI. NLI was associated with higher NIHSS score on admission, atherosclerosis, and cardiac embolism while LI was associated with small vessel disease.

**Table 2:** Characteristics of patients with LI and NLI based on DWI (n = 398)

	LI		NLI		P
		
	n	%	n	%	
Male	69	63.9	176	60.7	.64
Age (mean)	66.5 years		67.7 years		.46
NIHSS score on admission (mean)	3.3		5.1		.004
Atherosclerosis	9	8.3	54	18.7	.013
Cardiac embolism	6	5.6	81	28.0	< .001
Small vessel disease	67	62.0	4	1.4	< .001
LACS	66	61.1	45	15.5	*
PACS	19	17.6	144	49.8	*
TACS	0	0	46	15.8	*
POCS	23	21.3	55	18.9	*

* P < .001 (Pearson chi-square test)

LI: lacunar infarction

NLI: none-lacunar infarction

DWI: diffusion weighted imaging

NIHSS: National Institute of Health Stroke Scale

LACS: lacunar syndrome

PACS: partial anterior circulation syndrome

TACS: total anterior circulation syndrome

POCS: posterior circulation syndrome

Table 3 shows the association between OCSP score and DWI findings. NLI was detected in 40.5% of the patients with LACS. LI was detected among 11.7% of the patients with PACS.

**Table 3:** OCSP scores among patients with LI and NLI based on DWI (n = 398)

	LACS n (%)	TACS n (%)	PACS n (%)	POCS n (%)
LI	66 (59.5)	0 (0)	19 (11.7)	23 (29.5)
NLI	45 (40.5)	46 (100)	144 (88.3)	55 (70.5)

P < 0,001, Pearson chi-square

OCSP: Oxfordshire Community Stroke Project

LI: lacunar infarction

NLI: none-lacunar infarction

DWI: diffusion weighted imaging

LACS: lacunar syndrome

PACS: partial anterior circulation syndrome

TACS: total anterior circulation syndrome

POCS: posterior circulation syndrome

CT was performed among 561 (92%) of the patients. Acute ischemic lesions were detected among 162 (29%) patients. Among the latter 24(15%) had LI and 138 (85%) had NLI. Among patients with LI on CT, 4 (23.5%) patients had NLI on DWI. Among patients with NLI on CT, 2 (2.8%) patients had LI on DWI. CT was performed >24 hours after stroke onset among 52 patients with known onset of stroke. Acute ischemic lesions were detected among 22 (42%) of these patients.

Among patients with infarction in the posterior circulation who underwent DWI, 106 (96.4%) patients had DWI lesions. LI was found among 37 (34.9%) and NLI among 69 (65.1%) of these patients. Out of 106 patients with DWI lesions in the posterior circulation, 89 patients underwent CT examination on admission. CT showed acute ischemic lesion among 24 (27.0%) of these patients. LI was found in 4 (16.7%) and NLI in 20 (83.3%) of these patients on CT.

## Discussion

The main result was that patients presenting with neurological deficits suggestive of small vessel disease often had ischemic lesions on DWI associated with other etiologies such as atherosclerosis or cardiac embolism. Thus, lacunar syndrome was associated with none-lacunar infarction among 40.5% of the patients on DWI. This is in accordance with another study based on DWI which showed that clinical examination is not sufficient to differentiate lacunar from none-lacunar infarction [17]. However, that study was solely based on patients with lacunar infarction on DWI. Other studies have also shown that clinical examination misclassifies the ischemic lesion among a significant proportion of the patients, but these studies have relied heavily on CT [8,18].

CT on admission provides little extra information because only 29% of the CT scans as opposed to 95% of the DWI scans disclosed acute ischemic lesions. The low yield of CT compared with DWI is compatible with the findings in another study where 27% of CT scans and 85% of DWI scans showed acute ischemic lesions among patients with clinical acute cerebral infarction [12]. Furthermore, one out of four patients with lacunar infarction on CT had none-lacunar infarction on DWI. Thus a significant proportion of the patients with CT findings suggestive of small vessel disease may have another underlying etiology which needs appropriate investigation. The yield of CT is lowest in the early phase of cerebral infarction. However, acute ischemic lesions on CT were detected only among 42% of the patients when performed more than 24 hours after stroke onset in our study.

Most patients with anterior circulation syndrome (partial and total) had none-lacunar infarction on DWI. Furthermore, among patients with none-lacunar infarction on CT almost all had none-lacunar infarction on DWI. Thus, clinical presentation or CT suggestive of none-lacunar infarction are highly compatible with none-lacunar infarction on DWI.

The OCSP classification does not differentiate between lacunar and none-lacunar infarction in the posterior circulation. We found that 96.4% with posterior circulation infarctions had DWI lesions while CT showed acute ischemic lesions among only 27% of these patients. Furthermore, DWI is superior compared with CT as to differentiate between lacunar and none-lacunar infarctions in the posterior circulation.

Our study shows that clinical examination and CT poorly differentiates between lacunar infarction and none-lacunar infarction among patients with lacunar syndrome and posterior circulation syndrome. To guide further investigations among patients with lacunar or posterior circulation syndrome DWI is much superior to CT. Our results have implications for trials of neuroprotection and secondary preventive treatment where it may be important to distinguish between lacunar and none-lacunar infarctions.

Based on DWI we found that none-lacunar infarction was significantly associated with atherosclerosis and cardiac embolism while lacunar infarction was significantly associated with small vessel disease. This is compatible with the findings in another study [14]. Among our patients, 61.1% of the patients with lacunar infarction on DWI had lacunar syndrome. Another study showed that only 44.1% of the patients with lacunar infarction on DWI had lacunar syndrome [17]. Thus, only about one half of the patients with lacunar infarction on DWI present clinically with a lacunar syndrome.

One of the strengths of the present study is the prospective design including all consecutive patients with acute cerebral infarction admitted to a single centre reflecting real-life experience. Another strength is that a large proportion underwent DWI, especially among patients < 80 years. However, because not all had DWI Table 1 shows the characteristics of patients with and without DWI. Patients who had DWI were younger and had less severe infarctions. Another of the strengths of this study was that OCSP classification was performed within a week. Some other studies comparing OCSP and CT have had much longer inclusion interval after stroke onset [8]. A weakness of the present study was the delay between DWI and CT making a comparison between the yield of CT and DWI difficult. However, it is well-known that the yield of CT is much lower than DWI [12] compatible with the findings in the present study and the main objective of the present study was to compare clinical presentation with DWI.

## Conclusion

DWI is important among patients presenting with clinical symptoms suggestive of lacunar or posterior circulation syndrome in order to differentiate between lacunar and none-lacunar infarctions. On the other hand, there is good correlation between anterior circulation syndrome and none-lacunar infarction on DWI.

## Competing interests

The authors declare that they have no competing interests.

## Authors' contributions

HN performed analyses, collected data and wrote the manuscript. JB, TI, GM, and LT helped to draft the manuscript. UWA helped to draft the manuscript and collected data. All authors read and approved the final manuscript.

## Pre-publication history

The pre-publication history for this paper can be accessed here:


